# IVF outcomes of women with discrepancies between age and serum anti-Müllerian hormone levels

**DOI:** 10.1186/s12958-019-0498-3

**Published:** 2019-07-16

**Authors:** Bingqian Zhang, Yueru Meng, Xiao Jiang, Chao Liu, Huihui Zhang, Linlin Cui, Zi-Jiang Chen

**Affiliations:** 1 Center for Reproductive Medicine, Shandong Provincial Hospital Affiliated to Shandong University, Jingliu Road 157, Jinan, 250001 China; 2 National Research Center for Assisted Reproductive Technology and Reproductive Genetics, Jinan, China; 3 The Key laboratory of Reproductive Endocrinology (Shandong University), Ministry of Education, Jinan, China; 4 Shandong Provincial Clinical Medicine Research Center for Reproductive Health, Jinan, China; 5 Shandong Provincial Key Laboratory of Reproductive Medicine, No.157 Jingliu Road, Jinan, 250001 China; 6 Center of Reproductive Medicine, Linyi People,s Hospital, Linyi, China; 7 Center for Reproductive Medicine, Ren Ji Hospital, School of Medicine, Shanghai Jiao Tong University, Shanghai, China; 8 Shanghai Key Laboratory for Assisted Reproduction and Reproductive Genetics, Shanghai, China

**Keywords:** Advanced age, AMH, IVF, Live birth, Ovarian reserve

## Abstract

**Background:**

To determine the effects of age and the serum anti-Müllerian hormone (AMH) level on in vitro fertilization (IVF) outcomes, especially among young women with low serum AMH levels and older women with high AMH levels.

**Methods:**

This study was a cohort study in which a total of 9431 women aged 20–51 years who were undergoing their first IVF cycles were recruited. Ovarian response parameters included the number of retrieved oocytes, the number of 2 pronuclear zygotes (2PN), and the frequency of good-quality embryos (GQE). Pregnancy outcomes included the clinical pregnancy rate (CPR), live birth rate (LBR), miscarriage rate (MR), and cumulative CPR and LBR (CCPR and CLBR).

**Results:**

Among women under 35 years of age, the ovarian response, CPR, CCPR, LBR and CLBR (*p* < 0.01) were significantly lower in the low-AMH group than in the average-AMH and high-AMH groups. In women above 35 years of age, the ovarian response, CPR, CCPR and CLBR (*p* < 0.01) were significantly higher in the average-AMH and low-AMH groups. The LBR in the older high-AMH group was significantly higher (37.45% vs 20.34%, *p* < 0.01) than that in the older low-AMH group, but there was no difference (37.45% vs 32.46%, *p* = 0.11) compared with the older average-AMH group. When there was a discrepancy between age and the AMH level, the young low-AMH group showed a poorer ovarian response but a better CPR (58.01% vs 49.44%, *p* < 0.01) and LBR (48.52% vs 37.45%, *p* < 0.01) than the older high-AMH group. However, the CCPR (65.37% vs 66.11%, *p* = 0.75) and CLBR (56.35% vs 52.89%, *p* = 0.15) between the two groups were comparable. The conservative CLBR in the two discrepancy groups increased until the third embryo transfer and reached a plateau thereafter.

**Conclusion(s):**

Even with a relatively low AMH level, young women still had better pregnancy outcomes following IVF than older women. However, increasing the AMH level improves the cumulative outcomes of the older group to a comparable level through a notable and superior ovarian response.

**Electronic supplementary material:**

The online version of this article (10.1186/s12958-019-0498-3) contains supplementary material, which is available to authorized users.

## Background

The ovarian reserve refers to both the quantity and quality of oocytes and gradually decreases with increasing age in women of reproductive age [1].

Anti-mullerian hormone (AMH) is a glycoprotein of the transforming growth factor-β (TGF-β) family and is secreted primarily by the granulosa cells (GCs) of preantral and small antral follicles [2, 3]. It can inhibit the transition of the primordial follicle to the primary follicle and negatively regulate follicle growth [4]. The AMH level is strongly correlated with age [5] and the antral follicle count (AFC) [6] and is therefore an indicator of fecundity. Cumulative evidence suggests the strongly predictive value of the serum AMH level for ovarian response [7–9] and a modest predictive value for live birth [10, 11](in patients undergoing in vitro fertilization (IVF). It is well accepted that young women with a high AMH level perform notably well in terms of fecundity, while women of advanced age and with a low AMH level have poor IVF outcomes [12] .

However, due to high individual heterogeneity [13], a discrepancy exists between the two indicators in some patients, namely, with young women of reproductive age who have lower AMH levels and with those of advanced age who have higher AMH levels. These discrepancies confuse both physicians and patients during clinical counselling. A few studies on women within specific age groups have provided some insight. In women over 40 years old, the basal serum AMH level shows a higher correlation with ovarian response and the pregnancy rate with IVF treatment [14]. However, another study demonstrates that in women with very low (< 0.5 ng/ml) AMH levels, the pregnancy rate after IVF is significantly affected by chronological age [15]. Nevertheless, no study that has systemically assessed the IVF outcomes, namely, the clinical pregnancy and live birth rates, of women with a mismatch between chronological and biological age.

In the present study, we aimed to clarify the IVF outcomes, including the live birth rate (LBR) and cumulative live birth rate (CLBR), of women with discrepancies between their AMH levels and age, namely, young women with low AMH levels and older women with high AMH levels.

## Methods

### Study design and population

This study was a single-center retrospective cohort study that consecutively included women who underwent their first IVF cycles in Center for Reproductive Medicine, Shandong University, from March 2013 to June 2014. Of 9431 women, 7283 women of reproductive age were young (< 35 yr), and the other 2148 were at an advanced age (≥35 yr). In the young group, participants with low AMH levels (the 0-25th percentage, 0.01–1.32 ng/ml) were set as the exposed group (young low-AMH group, *n* = 1819), while those with medium AMH levels (the 25-75th percentage, 1.32–3.99 ng/ml) and high AMH levels (the 75-100th percentage, 3.99–22.05 ng/ml) were set as the unexposed group (young average-AMH group, *n* = 3642; young high-AMH group, *n* = 1822). In the advanced-aged group, women with high (the 75-100th percentage, 2.41–22.05 ng/ml) AMH levels were set as exposed group (older high-AMH group, *n* = 537), and women with medium (the 25-75th percentage, 0.63–2.41 ng/ml) AMH levels and low (the 0-25th percentage, 0.01–0.62 ng/ml) were set as the unexposed (older average-AMH group, *n* = 1074; older low-AMH group, *n* = 537) groups, respectively. Women who had eggs frozen or used donated eggs were excluded.

### Serum AMH assay

The serum AMH levels were measured before the first ovarian stimulation cycle. We excluded the factors which that may affect the AMH level, such as pregnancy, smoking and combined oral contraceptives. All blood samples were separated into aliquots within 2 h after collection and frozen at − 80 °C until use. Tests to determine the serum AMH levels were performed in batches using enzyme-linked immunosorbent assay (ELISA) (Ansh Labs, Webster, USA). The intra- and inter-assay coefficients of variation were below 10%.

### IVF and fresh embryo transfer (ET)

Different ovarian stimulation protocols were applied in this study and included the natural cycle, gonadotrophin-releasing hormone (GnRH) agonist long protocol [16], and flexible GnRH antagonist protocol [17], which have been described in detail previously. Briefly, for GnRH short protocols, the women received GnRH agonist (Diphereline, Ipsen) 0.1 mg daily starting on day 2 or 3 of the menstrual cycle. Recombinant FSH (Gonal-F, Merk Serono) was started after 2 days. The dosage was adjusted according to the ovarian response. Cycle monitoring was performed by ultrasonography and serum sex steroid hormone measurements. Ovulation triggering was implemented with a dose of 4000 to 8000 IU urinary human chorionic gonadotropin (HCG) when two or more follicles measured 18 mm. Collected oocytes were inseminated through IVF or ICSI. The later was performed when a total sperm count of < 500 000 after gradient centrifugation [18]. Embryos were cultured up to day 3 or day 5, and then, one or two embryos were transferred under ultrasound guidance. Intramuscular progesterone at a daily dose of 80 mg or dydrogesterone tablets (Duphaston) 10 mg two times daily were used by injection or orally for luteal phase support, from the day of oocyte retrieval until 10 weeks after conception.

### Frozen-thawed embryo transfer

Surplus embryos were cryopreserved by vitrification. Endometrial preparation was performed through either a natural cycle, an artificial cycle, or ovulation induction cycles. The natural cycle was applied to women with regular menstruation and normal ovulation in the B-ultrasound (US). The natural cycle was monitored by US. The artificial cycle was used for women with oligo/anovulation. Oral estradiol valerate (Progynova, Delpharm Lille) was administered for endometrial preparation. The protocol was the same as that described in our previous study [17]. For women in whom a satisfactory endometrium was not achieved using the above two protocols, we performed ovulation induction using low-dose HMG as an alternative. When the endometrial thickness reached 8 mm, intramuscular progesterone was added at a dose of 80 mg per day. In addition, one or two frozen embryos (day 5 or day 3) were thawed and transferred. The luteal-phase support continued until 10 weeks after conception.

### IVF outcomes

The AFC (antral follicle count) was defined using a 2D technique. Clinical pregnancy was considered as one or more gestational sacs visualized by ultrasound examination [17]. Miscarriage was considered as any spontaneous or therapeutic pregnancy loss during clinical pregnancy [17]. The inclusion of several treatment cycles for one subject the analysis would lead to bias. In our study, only the first treatment cycle was considered. Thus, the starting point for the LBR and CLBR was the first live birth in the first treatment cycle, and the follow-up examinations were ended when all fresh and frozen embryos derived from the first treatment cycle were transferred. Live birth was defined as delivery of at least one viable infant with a gestational age equal to or greater than 28 weeks [17]. The LBR per transfer was calculated by dividing the number of newborns by the transfer times [17]. Cumulative live birth was defined as the occurrence of live birth after the transfer of all fresh and frozen embryos derived from the first stimulation cycle. Each subject was included only once in the analysis.

Since not all the participants had used all of their embryos within the cycle by the time of the data collection, conservative and optimistic CLBRs were calculated as well. The conservative estimates assumed that women who did not return for frozen ET would not have the outcome of a live birth [19]. The 95% confidence interval (CI) was calculated using the standard error from the binomial distribution. The optimistic CLBR assumed that women who did not return for frozen ET would have similar outcomes to those who continued with frozen-thawed ET [19]. The pointwise optimal estimates and 95% CIs were assessed by the Kaplan–Meier estimate.

### Statistical analysis

Categorical data were represented as percentages and frequencies. Continuous data are expressed as the mean ± standard deviation (SD) and were tested for normality using the Kolmogorov-Smirnov test.

A 2 × 3 factorial analysis was conducted to test the effects of age and AMH and their interaction value. *P* < 0.05 was considered to be statistically significant. Pairwise comparisons were performed when the interaction *p* value was < 0.05. In women with age and AMH discrepancies, chi-square test or Fisher’s exact test was used for categorical data comparisons, and data with normal distributions were compared between groups using Student’s t-test. *P* < 0.05 was considered to be statistically significant. We used SPSS 23.0 for statistical analysis (SPSS Inc., USA).

Binary logistic regression was used to estimate associations between the miscarriage rate (MR) and age and AMH. *p* < 0.05 was considered to be statistically significant. The conservative and optimistic cumulative LBR estimation in advanced-aged women were calculated and graphed by Stata13.1.

## Results

The baseline characteristics are shown in Table 1. Among the young women (< 35 yr), subjects in the low-AMH group were older (29.46 ± 3.18 yr. vs 28.74 ± 3.16 yr. vs 28.26 ± 3.10 yr) and had a lower AFC (10.52 ± 4.78 vs 14.46 ± 5.80 vs 19.78 ± 9.41, *p* < 0.01 for all pairwise comparisons) than those in the average-AMH and high-AMH groups. Regarding the fertility history of the young women, the low-AMH group had a higher previous conception rate (41.67% vs 39.07% vs 34.69%, overall effect of AMH, *p* < 0.01) and POI (5.22% vs 0.41% vs 0, *p* < 0.01 for pairwise comparisons with the average- and high-AMH groups, respectively) but a lower PCOS rate (2.25% vs 7.36% vs 23.49%, *p* < 0.01 for all pairwise comparisons) and male factor infertility rate (30.90% vs 36.55% vs 37.87%, *p* < 0.01 for pairwise comparisons with the average- and high-AMH groups, respectively). The frequency of tubal-factor infertility in the low-AMH group was significantly higher than that in the high-AMH group (66.08% vs. 60.21%, *p* < 0.01), but there was no difference with the average-AMH group (66.08% vs. 64.74%, *p* = 0.7) (Table 1, Additional file 1: Table S1 and Table S2).

**Table 1:** Characteristics of the Patients at Baseline According Age and AMH Level

Characteristic	Age<35 yrs			Age ≥ 35 yrs		
	young low-AMH (*N* = 1819)	young average-AMH (*N* = 3642)	young high-AMH (*N* = 1822)	older low-AMH (*N* = 537)	older average-AMH (*N* = 1074)	older high-AMH (*N* = 537)
AMH range[%(ng/ml)]	0-25th quartile (0.01–1.32)	25th–75th quartile (1.32–3.99)	75th–100th quartile (3.99–22.05)	0-25th quartile (< 0.01–0.62)	25th–75th quartile (0.63–2.41)	75th–100th quartile (2.41–22.05)
Age (yrs)	29.46 ± 3.18	28.74 ± 3.16	28.26 ± 3.10	38.67 ± 2.90	37.88 ± 2.57	37.12 ± 2.16
BMI (kg/m2)	23.12 ± 3.69	22.90 ± 3.42	22.82 ± 3.62	24.00 ± 3.15	24.11 ± 3.19	23.99 ± 3.29
AFC	10.52 ± 4.78^a,b^	14.46 ± 5.80^c^	19.78 ± 9.41	7.14 ± 2.93^d,e^	10.47 ± 5.38^f^	15.18 ± 7.04
Fertility history						
Previous conception[%(n/N)]	41.67(758/1819)^g^	39.07(1423/3642)	34.69(632/1822)	72.75(388/537)	74.77(803/1074)	66.85(359/537)
Concomitant infertility factors						
Tubal factors[%(n/N)]	66.08(1202/1819)^b^	64.74(2358/3642)^c^	60.21(1097/1822)	68.34(367/537)	71.79(771/1074)	70.58(379/537)
Ovulatory dysfunction[%(n/N)]						
PCOS[%(n/N)]	2.25(41/1819)^a,b^	7.36(268/3642)^c^	23.49(428/1822)	0.56(3/537)^e^	1.96(21/1074)^f^	10.80(58/537)
POI[%(n/N)]	5.22(95/1819)^a,b^	0.41(15/3642)	0(0/0)	8.39(45/537)^d,e^	1.96(21/1074)	0.19(1/537)
Male factors[%(n/N)]	30.90(562/1819)^a,b^	36.55(1331/3642)	37.87(690/1822)	22.91(123/537)	26.44(284/1074)	25.14(135/537)
Controlled Ovarian Hyperstimulation outcome						
Gonadotropin start dose (IU)	196.40 ± 48.96^a,b^	177.05 ± 44.02^c^	162.95 ± 42.41	230.48 ± 49.82^d,e^	218.14 ± 48.73^f^	197.69 ± 41.29
Total Gonadotropin dose (IU)	2130.27 ± 982.84^g^	1937.00 ± 778.37	1782.24 ± 762.80	2375.30 ± 1108.22	2286.36 ± 970.59	2140.51 ± 800.74
No. of days of ovarian stimulation	10.52 ± 2.02^g^	10.82 ± 1.78	10.94 ± 1.95	10.06 ± 2.28	10.41 ± 2.03	10.85 ± 1.93
Endometrial thickness on hCG trigger day(cm)	1.11 ± 0.20	1.13 ± 0.18	1.12 ± 0.18	1.02 ± 0.23^d,e^	1.07 ± 0.20	1.10 ± 0.20

*AMH* anti-Müllerian hormone, *BMI* body Mass Index, *AFC* antral follicle count

Continuous data was presented as mean ± standard deviation, and categorical variable was presented as percentile (number)

Significant subgroup differences (Bonferroni pairwise comparison, *p* < 0.05/3) are indicated by superscripts “a: low AMH vs. average AMH in women < 35 yrs”, “b: low AMH vs. high AMH in women < 35 yrs”, “c: average AMH vs. high AMH in women < 35 yrs”, “d: low AMH vs. average AMH in women ≥35 yrs”, “e significant difference for low AMH vs. high AMH in women ≥35 yrs”,“f significant difference for average AMH vs. high AMH in women ≥35 yrs” g: *p* < 0.05 was considered statistically significant difference for main effect

Among the women of advanced age (≥35 yr), the high-AMH group was younger (37.12 ± 2.16 yr. vs 37.88 ± 2.57 yr. vs 38.67 ± 2.90 yr) and had a higher AFC (15.18 ± 7.04 vs 10.47 ± 5.38 vs 7.14 ± 2.93, *p* < 0.01 for all pairwise comparisons) than average-AMH and low-AMH groups. The previous conception rate was lower in the high-AMH group (66.85% vs 74.77 vs 72.75%, overall effect of AMH, *p* < 0.01) than in the average- and low-AMH groups. As expected, the incidence of PCOS was higher in the high-AMH group (10.80% vs 1.96%. vs 0.56%, *p* < 0.01 for pairwise comparisons with the average- and low-AMH groups), but the incidence of POI was lower (0.19% vs 1.96% vs 8.39%, *p* < 0.01 compared with the low-AMH group and *p* = 0.07 compared with the average-AMH group).

### IVF outcome comparisons within age-stratified groups

The IVF outcomes of each age-stratified group are listed in Table 2, Additional file 1: Table S1 and Table S2, Additional file 2: Figure S1. In young group, the number of oocytes retrieved (9.15 ± 5.09 vs 13.13 ± 5.49 vs 16.04 ± 6.44, *p* < 0.01 for all pairwise comparisons), the number of 2 pronuclear zygotes (2PN, 5.32 ± 3.59 vs 7.88 ± 4.21 vs 9.31 ± 4.59, *p* < 0.01 for all pairwise comparisons), and the frequency of good-quality embryos (GQE, 2.42 ± 2.53 vs 3.50 ± 3.22 vs 4.08 ± 3.78, *p* < 0.01 compared with the average- and high-AMH groups, respectively) were significantly lower in low-AMH group than in the average-AMH and high-AMH groups. The clinical pregnancy rate (CPR, 58.01% vs 62.90% vs 66.33%, *p* < 0.01 for all pairwise comparisons), cumulative clinical pregnancy rate (CCPR, 65.37% vs 78.31% vs 83.15%, *p* < 0.01 for all pairwise comparisons), LBR (48.52% vs 54.65% vs 56.68%, *p* < 0.01 compared with the average- and high-AMH groups, respectively), and CLBR (56.35% vs 69.99% vs 72.99%, *p* < 0.01 for compared with the average- and high-AMH groups, respectively) were also decreased. It was shown that age, but not AMH, was the risk factor for miscarriage (Age: OR = 1.05, 95% CI = 1.02–1.08, *p* < 0.01; AMH: OR = 1.01, 95% CI = 0.97–1.05, *p* = 0.56, Additional file 1: Table S3).

**Table 2:** Outcomes of Pregnancy

Characteristic	Age<35 yrs			Age ≥ 35 yrs		
	young low-AMH	young average-AMH	young high-AMH	older low-AMH	older average-AMH	older high-AMH
	(*N* = 1819)	(*N* = 3642)	(*N* = 1822)	(*N* = 537)	(*N* = 1074)	(*N* = 537)
No. of oocytes retrieved(n)	9.15 ± 5.09^a,b^	13.13 ± 5.49^c^	16.04 ± 6.44	4.53 ± 3.25^d,e^	8.45 ± 4.39^f^	12.91 ± 5.73
No. of 2PN(n)	5.32 ± 3.59^a,b^	7.88 ± 4.21^c^	9.31 ± 4.59	2.70 ± 2.30^d,e^	5.15 ± 3.53^f^	7.79 ± 4.39
No. of GQE (cleavage stage) (n)	2.42 ± 2.53^a,b^	3.50 ± 3.22	4.08 ± 3.78	1.31 ± 1.54^d,e^	2.28 ± 2.36^f^	3.60 ± 3.32
IVF(n)	1180	2129	1158	405	722	370
ICSI(n)	447	1036	501	117	218	109
Fresh embryo transfer(n)	1510	2923	1138	396	905	426
Frozen embryo transfer(n)	722	2069	1443	135	475	383
CPR[%(n/N)]	58.01(1295/2232)^a,b^	62.90(3140/4992)^c^	66.33(1712/2581)	33.90(180/531)^d,e^	41.45(572/1380)^f^	49.44(400/809)
CCPR[%(n/N)]	65.37(1189/1819)^a,b^	78.31(2852/3642)^c^	83.15(1515/1822)	30.73(165/537)^d,e^	49.07(527/1074)^f^	66.11(355/537)
LBR [%(n/N)]	48.52(1083/2232)^a,b^	54.65(2728/4992)	56.68 (1463/2581)	20.34(108/531)^d,e^	32.46(448/1380)	37.45(303/809)
CLBR[%(n/N)]	56.35(1025/1819)^a,b^	69.99(2549/3642)	72.99(1330/1822)	20.11(108/537)^d,e^	39.29(422/1074)^f^	52.89(284/537)
Miscarriage rate [%(n/N)]	16.37(212/1295)	13.28(417/3140)	14.54(249/1712)	35.56(64/180)^d,e^	21.68(124/572)	21.50(86/400)

*2PN* 2 pronuclear, *GQE* good-quality embryo, *CPR* clinical pregnancy rate, *CCPR* cumulative clinical pregnancy rate, *LBR* live birth rate, *CLBR* cumulative live birth rate

Continuous data was presented as mean ± standard deviation, and categorical variable was presented as percentile (number)

Significant subgroup differences (Bonferroni pairwise comparison, *p* < 0.05/3) are indicated by superscripts “a: low AMH vs. average AMH in women < 35 yrs”, “b: low AMH vs. high AMH in women < 35 yrs”, “c: average AMH vs. high AMH in women < 35 yrs”, “d: low AMH vs. average AMH in women ≥35 yrs”, “e significant difference for low AMH vs. high AMH in women ≥35 yrs”,“f significant difference for average AMH vs. high AMH in women ≥35 yrs”

Among advance-aged groups, the number of oocytes retrieved (12.91 ± 5.73 vs 8.45 ± 4.39 vs 4.53 ± 3.25, *p* < 0.01 for all pairwise comparisons), the number of 2PN zygotes (7.79 ± 4.39 vs 5.15 ± 3.53 vs 2.70 ± 2.30, *p* < 0.01 for all pairwise comparisons), the frequency of GQE (3.60 ± 3.32 vs 2.28 ± 2.36 vs 1.31 ± 1.54, *p* < 0.01 for all pairwise comparisons) were significantly higher in the high-AMH group than in the low-AMH group. Pregnancy outcomes including the CPR (49.44% vs 41.45% vs 33.90%, *p* < 0.01 for all pairwise comparisons), CCPR (66.11% vs 49.07% vs 30.73%, *p* < 0.01 for all pairwise comparisons), and CLBR (52.89% vs 39.29% vs 20.11%, *p* < 0.01 for all pairwise comparisons) were also significantly higher in older women with high AMH levels. The frequency of LBR in the older high-AMH group was significantly higher (37.45% vs 20.34%, *p* < 0.01) than that in the young low-AMH group, but there was no difference (37.45% vs 32.46%, *p* = 0.11) compared with the average-AMH group. Moreover, the MR in the high-AMH group was significantly lower (21.50% vs 35.56%, *p* < 0.01) than that in the low-AMH group, while no difference (21.50% vs 21.68%, *p* = 0.97) was found compared with their average-AMH counterpart. Age, but not AMH, was correlated with MR in women of advanced age (Age: OR = 1.36, 95% CI = 1.26–1.49, *p* < 0.01; AMH: OR = 1.00, 95% CI = 0.91–1.10, *p* = 0.99) (Additional file 1: Table S3).

### IVF outcome comparisons between groups with discrepancies

When the IVF outcomes were compared between the two groups with discrepancies between age and the AMH level (the young low-AMH group and older high-AMH group), it was found that the number of oocytes retrieved (9.15 ± 5.09 vs 12.91 ± 5.73, *p* < 0.01), the number of 2PN zygotes (5.32 ± 3.59 vs 7.79 ± 4.39, *p* < 0.01), and the frequency of GQE (2.42 ± 2.53 vs 3.60 ± 3.32, *p* < 0.01) were significantly lower in the young low-AMH group than in the older high-AMH group. However, the CPR (58.01% vs 49.44%, *p* < 0.01) and LBR (48.52% vs 37.45%, *p* < 0.01) was significantly higher in the former. The CCPR (65.37% vs 66.11%, *p* = 0.75) and CLBR (56.35% vs 52.89%, *p* = 0.15) were comparable between both groups. No difference was found in MR between the groups (16.37% vs 21.50%, *p* < 0.01) (Table 3).

**Table 3:** Outcome in groups of women with discrepancies between age and serum anti-Müllerian hormone levels

	young low-AMH (*N* = 1819)	older high-AMH (*N* = 537)	p
No. of oocytes retrieved(n)	9.15 ± 5.09	12.91 ± 5.73	< 0.01*
No. of 2PN(n)	5.32 ± 3.59	7.79 ± 4.39	< 0.01*
No. of GQE (cleavage stage)(n)	2.42 ± 2.53	3.60 ± 3.32	< 0.01*
IVF(n)	1180	370	0.04
ICSI(n)	447	109	
Fresh embryo transfer(n)	1510	426	< 0.01*
Frozen embryo transfer(n)	722	383	
CPR[%(n/N)]	57.71(1295/2232)	49.44(400/809)	< 0.01*
CCPR[%(n/N)]	65.37(1189/1819)	66.11(355/537)	0.75
LBR [%(n/N)]	48.52(1083/2232)	37.45(303/809)	< 0.01*
CLBR[%(n/N)]	56.35(1025/1819)	52.89(284/537)	0.15
Miscarriage rate [%(n/N)]	16.37(212/1295)	21.50(86/400)	0.03*

*2PN* 2 pronuclear, *GQE* good-quality embryo, *CPR* clinical pregnancy rate, *CCPR* cumulative clinical pregnancy rate, *LBR* live birth rate, *CLBR* cumulative live birth rate

Continuous data was presented as mean ± standard deviation, and categorical variable was presented as percentile (number)

*p < 0.05 was set as significant difference

**Fig. 1:**
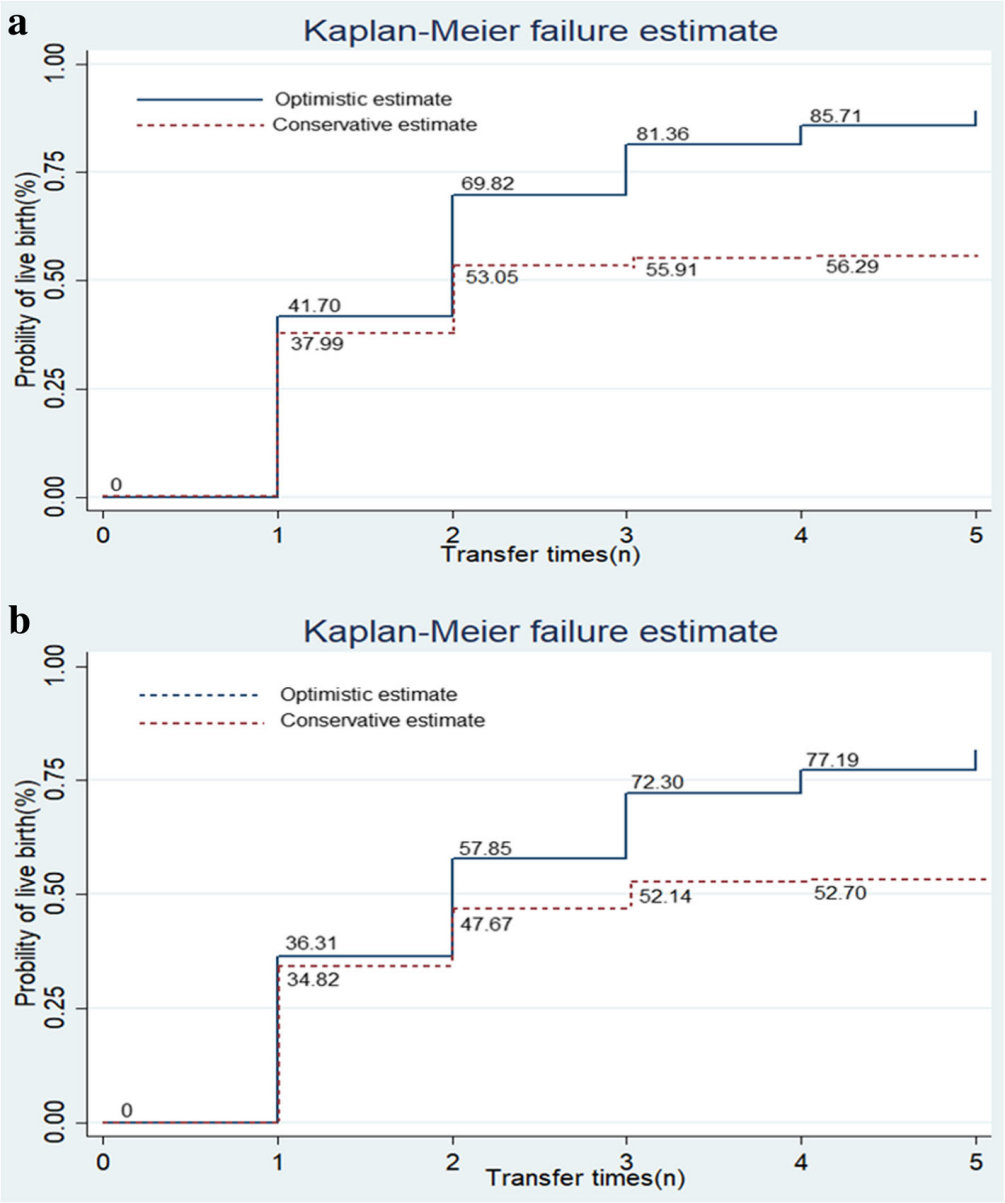
Optimistic and Conservative cumulative live birth rate in women with age and AMH discrepancies. **a** young women with low AMH level. **b** elder women with high AMH level. The conservative estimates assumed that women who did not return for embryo transfer would not have the outcome of live birth. The optimistic CLBR assumed that women did not return for embryo transfer would have similar outcome rates to those who continued frozen-thawed embryo transfer, which was made by the Kaplan–Meier estimate. The two curves showed the best and worst estimates of the women ≥35 years

The optimistic and conservative CLBRs of women in terms of age and the AMH level are presented in Fig. 1 and Additional file 1: Table S4. In the young women with low AMH levels, the optimistic CLBR increased along with the transfer time from 41.70% (95% CI: 39.37–44.12) for the first ET to 89.28% (95% CI: 81.58–94.76) for the fifth ET. In addition, the conservative CLBR increased from 37.99% (95% CI: 35.75–40.26) for the first ET to 55.91% (95% CI: 53.59–58.21) for the third ET. In the older women with high AMH levels, the optimistic CLBR increased from 36.31% (95% CI: 32.32–40.63) after the first ET to 81.75% (95% CI: 71.19–90.23) after the fifth ET. In addition, the conservative CLBR increased from 34.82% (95% CI: 30.79–39.02) after the first ET to 52.14% (95% CI: 47.82–56.44) after the third ET. After the third ET, the conservative CLBR in both discrepancy groups reached a plateau regardless of whether more ETs were performed. We calculated the ROC curves for prediction of live birth and cumulative live birth in low-AMH young and high-AMH elder groups. However, the predictive value was poor , which indicated that AMH had a significant association with live birth but was a poor independent predictor for live birth (Additional file 3: Figure S2).

## Discussion

The present study showed that in women with a discrepancy between age and the AMH level, the ovarian response was positively correlated with the serum AMH level, but the pregnancy outcome did not show a similar trend. The young low-AMH group showed a higher CPR and LBR than the older high-AMH group but a similar CCPR and CLBR to this group. Regarding the CLBR of women with a discrepancy in age and the AMH level, a plateau was reached after three ET times.

In our study, the AMH level was positively associated with the number of oocytes retrieved in both the young and advance-aged groups, which is consistent with the results of a previous study [6]. The number of oocytes retrieved was a robust surrogate outcome for clinical success [20, 21]. This may be due to the elevated number of GQE, which increases the number of ET attempts. Our analysis suggested that for young patients with low ovarian reserve, at least 3 ET attempts would optimize the IVF outcome. This result provides additional encouragement and evidence for this subgroup to undergo more trials. This finding was consistent with that of our previous study on POR patients [22] but differed slightly with the findings of Xu et al. [23], whose study suggested a maximum of four attempted embryo transfer cycles. However, for older women, although a high AMH level indicated the obtaining of more oocytes, the CLBR also reached a plateau after the third embryo transfer according to our data. The decreased competency of the oocyte may be the key mechanism.

In women of advanced age, the LBR did not improve even when the AMH level was prominently high. Compared with young women with low AMH levels, older women with high AMH levels still showed a decreased CPR and LBR, as well as an increased MR. The underlying reason may be the age-related deterioration of oocyte competence [24, 25] in advanced-aged women. The impairment of mitochondrial function [26] and the high production of reactive oxygen species (ROS) [27–29] in aged GCs are suggested to be the mechanisms. The physiological changes that occur with aging could increase mitochondrial (mt) DNA instability, decrease mitochondrial biogenesis, induce DNA damage in oocytes, and cause the disassembly of oocyte spindles, which consequently decreases the oocyte quality [26, 30]. According to the present data, age was more important than the AMH level in the assessment of outcomes per transfer and was the only risk factor for miscarriage in both age groups. This conclusion was inconsistent with that of another recent study, which suggested both age and the AMH level as risk factors of miscarriage [31]. This difference might be attributed to different inclusion criteria (the aforementioned study included only fresh ET cycles) and the younger age of the study participants.

To our knowledge, there are few relevant studies that are specific to women with a discrepancy between age and the ovarian reserve [32]. The present study fills this knowledge gap and provides important evidence for clinical counseling before IVF treatment. In addition, the CLBR following the transfer of all fresh and frozen embryos was set as the primary outcome, which was the most meaningful for the patients. However, there were still some limitations for our study. First, although we included IVF patients only, which limited the frequency of male-factor infertility to some extent, there were still other confounders, such as heterogeneity caused by different ovarian stimulating settings and endometrial receptivity. Second, approximately 5% of patients had frozen embryos remaining, which might have influenced the outcome. To address this, we estimated the optimistic and conservative CLBRs. The final outcome was supposed to fall between them. In our research, the CPR was higher compared to official monitored paper in the European counties. However, it was consisted with our previous RCT researches, which was acknowledged in The New England Journal of Medicine. The reason was unclear and needed investigation [17, 33].

## Conclusion

In conclusion, even with a poor ovarian reserve, young women still had more favorable pregnancy outcomes following IVF than women of advanced age. A high AMH level could not improve the outcomes per transfer for older women but could improve the cumulative outcomes to a level that was comparable to the outcomes of their young counterparts through more attempts at ET. Within three ET cycles, the chance of pregnancy improved. This is important in counseling patients regarding their expectations.

## Additional files

Additional file 1: **Table S1.** 2*3 factorial analysis outcomes for assigned to age and AMH exposure. **Table S2.** Pairwise comparison analysis outcomes for assigned to age and AMH exposure. **Table S3.** Occurrence of miscarriage according to age and AMH. **Table S4.** Conservative and Optimistic Cumulative Live Birth Rate (CLBR) by Transfer Time Increase. (DOCX 43 kb)

Additional file 2: **Figure S1.** Clinical pregnancy (a), cumulative clinical pregnancy (b), live birth (c) and cumulative live birth (d) and miscarriage (e) rate in women with reproductive age. Black bars: women less than 35 years old. Shaded bars: women above 35 years old. CPR: clinical pregnancy rate; CCPR: cumulative clinical pregnancy rate; LBR: live birth rate; CLBR: cumulative live birth rate. (TIF 131 kb)

Additional file 3: **Figure S2.** AMH ROC curves for LBR (a) and CLBR (b) in young women with low AMH, and for LBR (c) and CLBR (d) in older women with high AMH. CPR: clinical pregnancy rate; CCPR: cumulative clinical pregnancy rate; LBR: live birth rate; CLBR: cumulative live birth rate. (TIF 147 kb)

## Data Availability

The datasets used and/or analysed during the current study are available from the corresponding author on reasonable request.

## Abbreviations

2PN: 2 pronuclear zygotes
AFC: Antral follicle count
AMH: Anti-Mullerian hormone
CCPR: Cumulative clinical pregnancy rate
CLBR: Cumulative live birth rate
CPR: Clinical pregnancy rate
ET: Embryo transfer
GCs: Granulosa cells
GIUT: Gamete intra-uterine transfer
GnRH: Gonadotrophin-releasing hormone
GQE: Good quality embryos
HCG: Human chorionic gonadotropin
ICSI: Intracytoplasmic sperm injection
IVF: in vitro fertilization
LBR: Live birth rate
MR: Miscarriage rate
PGD: Preimplantation genetic diagnosis
TGF-β: Transforming growth factor-β
