# Supplementary material for: IVF outcomes of women with discrepancies between age and serum anti-Müllerian hormone levels
Source: Reprod Biol Endocrinol. 2019 Jul 16;17:58. doi: 10.1186/s12958-019-0498-3 (PMC6636016; doi:10.1186/s12958-019-0498-3)
Supplement: Supplementary file 1 — Table S1. 2*3 factorial analysis outcomes for assigned to age and AMH exposure. Table S2. Pairwise comparison analysis outcomes for assigned to age and AMH exposure. Table S3. Occurrence of miscarriage according to age and AMH. Table S4. Conservative and Optimistic Cumulative Live Birth Rate (CLBR) by Transfer Time Increase. (DOCX 43 kb) [file 12958_2019_498_MOESM1_ESM.docx]

Additional file 1: Table S1 2*3 factorial analysis outcomes for assigned to age and AMH exposure

| **Outcome** | AMH effect over age level |  | age effect over AMH level | Interaction P value |
| --- | --- | --- | --- | --- |
| BMI (kg/m2) | 0.59 |  | <0.01* | 0.51 |
| AFC（n） | <0.01* |  | <0.01* | 0.02* |
| Fertility history |  |  |  |  |
| Previous conception[%(n/N)] | <0.01* |  | <0.01* | 0.24 |
| Tubal factors[%(n/N)] | <0.01* |  | 0.13 | 0.05* |
| Ovulatory dysfunction[%(n/N)] |  |  |  |  |
| PCOS[%(n/N)] | <0.01* |  | <0.01* | <0.01* |
| POI[%(n/N)] | <0.01* |  | <0.01* | <0.01* |
| Male factors[%(n/N)] | <0.01* |  | 0.07 | 0.05* |
| Controlled Ovarian Hyperstimulation outcome |  |  |  |  |
| Gonadotropin Start dose (IU) | <0.01* |  | <0.01* | 0.01* |
| Total Gonadotropin dose (IU) | <0.01* |  | <0.01* | 0.1 |
| No. of days of ovarian stimulation(n) | <0.01* |  | <0.01* | 0.1 |
| Endometrial thickness on hCG trigger day(mm) | <0.01* |  | <0.01* | <0.01* |
| No. of oocytes retrieved(n) | <0.01* |  | <0.01* | <0.01* |
| No. of 2PN(n) | <0.01* |  | <0.01* | <0.01* |
| No. of GQE (cleavage stage) (n) | <0.01* |  | <0.01* | <0.01* |
| Pregnancy outcome |  |  |  |  |
| CPR[%(n/N)] | <0.01* |  | <0.01* | 0.03* |
| CCPR[%(n/N)] | <0.01* |  | <0.01* | <0.01* |
| LBR [%(n/N)] | <0.01* |  | <0.01* | 0.01* |
| CLBR[%(n/N)] | <0.01* |  | <0.01* | <0.01* |
| Miscarriage rate [%(n/N)] | 0.03* |  | <0.01* | 0.01* |

AMH: anti-Müllerian hormone, BMI: body Mass Index, AFC: antral follicle count

2PN:2 pronuclear; GQE: good-quality embryo; CPR: clinical pregnancy rate; CCPR: cumulative clinical pregnancy rate ;LBR: live birth rate; CLBR: cumulative live birth rate

* p<0.05 was considered statistically significant difference for main effect

Additional file 1: Table S2 Pairwise comparison analysis outcomes for assigned to age and AMH exposure

| **Outcome** | AMH effect over age level | | | | | | |  | age effect over AMH level | | |
| --- | --- | --- | --- | --- | --- | --- | --- | --- | --- | --- | --- |
|  | <35 yrs | | |  | ≥35 yrs | | |  | low | average | high |
|  | low vs. average | average vs. high | low vs. high |  | low vs. average | average vs. high | low vs. high |  | young vs. elder | young vs. elder | young vs. elder |
| AFC | <0.01* | <0.01* | <0.01* |  | <0.01* | <0.01* | <0.01* |  | <0.01^#^ | <0.01^#^ | <0.01^#^ |
| Fertility history |  |  |  |  |  |  |  |  |  |  |  |
| Tubal factors[%(n/N)] | 0.7 | <0.01* | <0.01* |  | 0.43 | 0.95 | 0.83 |  | 0.33 | <0.01^#^ | <0.01^#^ |
| Ovulatory dysfunction[%(n/N)] |  |  |  |  |  |  |  |  |  |  |  |
| PCOS[%(n/N)] | <0.01* | <0.01* | <0.01* |  | 0.7 | <0.01* | <0.01* |  | 0.2 | <0.01^#^ | <0.01^#^ |
| POI[%(n/N)] | <0.01* | 0.63 | <0.01* |  | <0.01* | 0.04* | <0.01* |  | <0.01^#^ | <0.01^#^ | 0.78^#^ |
| Malefactors[%(n/N)] | <0.01* | 0.71 | <0.01* |  | 0.96 | 0.69 | 0.96 |  | <0.01^#^ | <0.01^#^ | <0.01^#^ |
| COH outcome |  |  |  |  |  |  |  |  |  |  |  |
| Gonadotropin start dose(IU) | <0.01* | <0.01* | <0.01* |  | <0.01* | <0.01* | <0.01* |  | <0.01^#^ | <0.01^#^ | <0.01^#^ |
| Endometrial thickness on hCG trigger day(mm) | 0.02* | 0.27 | 0.7 |  | <0.01* | 0.02* | <0.01* |  | <0.01^#^ | <0.01^#^ | 0.03 |
| Pregnancy outcome |  |  |  |  |  |  |  |  |  |  |  |
| No. of oocytes retrieved(n) | <0.01* | <0.01* | <0.01* |  | <0.01* | <0.01* | <0.01* |  | <0.01^#^ | <0.01^#^ | <0.01^#^ |
| No. of 2PN(n) | <0.01* | <0.01* | <0.01* |  | <0.01* | <0.01* | <0.01* |  | <0.01^#^ | <0.01^#^ | <0.01^#^ |
| No. of GQE (cleavage stage)(n) | <0.01* | 0.03 | <0.01* |  | <0.01* | <0.01* | <0.01* |  | <0.01^#^ | <0.01^#^ | <0.01^#^ |
| Pregnancy outcome |  |  |  |  |  |  |  |  |  |  |  |
| CPR[%(n/N)] | <0.01* | <0.01* | <0.01* |  | <0.01* | <0.01* | <0.01* |  | <0.01^#^ | <0.01^#^ | <0.01^#^ |
| CCPR[%(n/N)] | <0.01* | <0.01* | <0.01* |  | <0.01* | <0.01* | <0.01* |  | <0.01^#^ | <0.01^#^ | <0.01^#^ |
| LBR[%(n/N)] | <0.01* | 0.98 | <0.01* |  | <0.01* | 0.11 | <0.01* |  | <0.01^#^ | <0.01^#^ | <0.01^#^ |
| CLBR[%(n/N)] | <0.01* | 0.07* | <0.01* |  | <0.01* | <0.01* | <0.01* |  | <0.01^#^ | <0.01^#^ | <0.01^#^ |
| MR[%(n/N)] | 0.03* | 0.55 | 0.44 |  | <0.01* | 0.97 | <0.01* |  | <0.01^#^ | <0.01^#^ | <0.01^#^ |

AMH: anti-Müllerian hormone; COH: Controlled Ovarian Hyperstimulation

2PN:2 pronuclear; GQE: good-quality embryo; CPR: clinical pregnancy rate; CCPR: cumulative clinical pregnancy rate; LBR: live birth rate; CLBR: cumulative live birth rate

Bonferroni pairwise comparison are indicated by superscripts：*p<0.017(0.05/3) was considered statistically signiﬁcant difference; ^#^ p<0.025(0.05/2) was considered statistically signiﬁcant difference

Additional file 1: Table S3 Occurrence of miscarriage according to age and AMH

|  | p | OR（95%CI） |
| --- | --- | --- |
| Age＜35 yrs |  |  |
| Age（yrs） | ＜0.01* | 1.05(1.02-1.08) |
| AMH(ng/ml) | 0.56 | 1.01(0.97-1.05) |
| Age≥35 yrs |  |  |
| Age（yrs） | ＜0.01* | 1.36(1.26-1.49) |
| AMH(ng/ml) | 0.99 | 1.00(0.91-1.10) |

Binary logistic regression was applied to test the correlation with age and AMH both in young and advanced women.

AMH: Antimüllerian hormone, OR:odds ratio, CI: confidence interval

*p＜0.05 was considered to be statistically significant

Additional file 1: Table S4 Conservative and Optimistic Cumulative Live Birth Rate (CLBR) by Transfer Time Increase

| Group | Transfer times | No. of women | No. of women with at least one live birth | Conservative CLBR [%(95CI)] | Optimistic CLBR [%(95CI)] |
| --- | --- | --- | --- | --- | --- |
| Young low-AMH | 0 | 1819^a^ | 0 | 0 | 0 |
|  | 1 | 1657 | 691 | 37.99(35.75,40.26) | 41.70(39.37,44.12) |
|  | 2 | 568 | 274 | 53.05(50.73,55.37) | 69.82(67.12,72.49) |
|  | 3 | 136 | 52 | 55.91(53.59,58.21) | 81.36(78.30,84.23) |
|  | 4 | 30 | 7 | 56.29(53.98,58.59) | 85.71(81.87,89.10) |
|  | 5 | 4 | 1 | 56.35(54.03,58.54) | 89.28(81.58,94.76) |
| Older high-AMH | 0 | 537^b^ | 0 | 0 | 0 |
|  | 1 | 515 | 187 | 34.82(30.79,39.02) | 36.31(32.32,40.63) |
|  | 2 | 204 | 69 | 47.67(43.38,51.99) | 57.85(52.95,62.85) |
|  | 3 | 70 | 24 | 52.14(47.82,56.44) | 72.30(66.49,77.85) |
|  | 4 | 17 | 3 | 52.70(48.38,56.99) | 77.19(70.04,83.68) |
|  | 5 | 5 | 1 | 52.89(48.57,57.18) | 81.75(71.19,90.23) |

The 95% confidence intervals (95%CI) of conservative estimation were calculated using standard errors from the binomial distribution.

The optimistic estimates and 95%CI were assessed by the Kaplan–Meier estimate.

^a^ In low-AMH young group, 162 women didn’t take the oocyte.

^b^ In high-AMH elder group, 22 women didn’t take the oocyte.
