# Supplementary figures and images for: IVF outcomes of women with discrepancies between age and serum anti-Müllerian hormone levels
Source: Reprod Biol Endocrinol. 2019 Jul 16;17:58. doi: 10.1186/s12958-019-0498-3 (PMC6636016; doi:10.1186/s12958-019-0498-3)

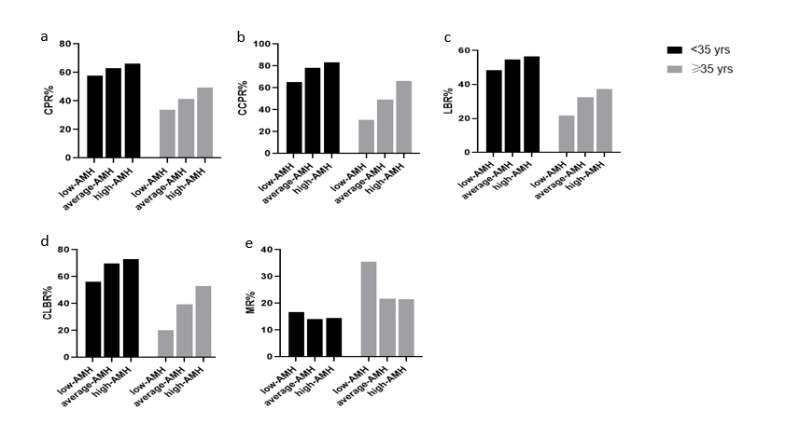

Supplement: Supplementary file 2 — Figure S1. Clinical pregnancy (a), cumulative clinical pregnancy (b), live birth (c) and cumulative live birth (d) and miscarriage (e) rate in women with reproductive age. Black bars: women less than 35 years old. Shaded bars: women above 35 years old. CPR: clinical pregnancy rate; CCPR: cumulative clinical pregnancy rate; LBR: live birth rate; CLBR: cumulative live birth rate. (TIF 131 kb) [file 12958_2019_498_MOESM2_ESM.tif]

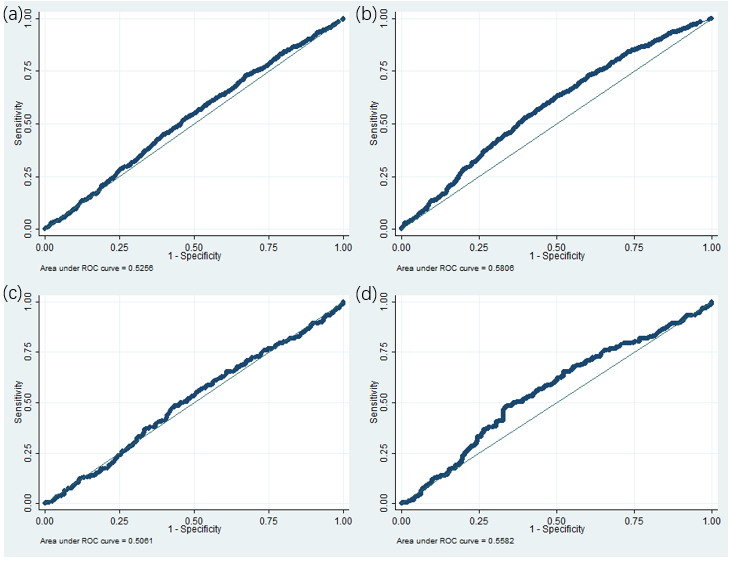

Supplement: Supplementary file 3 — Figure S2. AMH ROC curves for LBR (a) and CLBR (b) in young women with low AMH, and for LBR (c) and CLBR (d) in older women with high AMH. CPR: clinical pregnancy rate; CCPR: cumulative clinical pregnancy rate; LBR: live birth rate; CLBR: cumulative live birth rate. (TIF 147 kb) [file 12958_2019_498_MOESM3_ESM.tif]
